# Listeria monocytogenes: the silent assassin

**DOI:** 10.1099/jmm.0.001800

**Published:** 2024-03-20

**Authors:** Emily T. Fotopoulou, Claire Jenkins, Anaïs Painset, Corinne Amar

**Affiliations:** 1 UK Health Security Agency, Gastrointestinal Bacteria Reference Unit, 61 Colindale Avenue, London, NW9 5EQ, UK; 2 National Institute of Health & Care Research, Health Protection Research Unit in Gastrointestinal Infections, University of Liverpool, Liverpool, L3 5TR, UK; 3 School of Life Sciences and Department of Statistics, University of Warwick, Coventry, CV4 7AL, UK

**Keywords:** *Listeria monocytogenes*, clinical outcomes, virulence factors, microbiology, epidemiology

## Abstract

Listeriosis is a foodborne infection in humans caused by *Listeria monocytogenes.* Consumption of contaminated food can lead to severe infection in vulnerable patients, that can be fatal. Clinical manifestations include sepsis and meningitis, and in pregnancy-associated infection, miscarriage and stillbirth. Diagnosis is confirmed by culture and identification of the pathogen from blood, cerebrospinal fluid, vaginal swab, placenta or amniotic fluid. Treatment regimens recommend amoxicillin, ampicillin or an aminoglycoside. Virulence factors mediate bacterial adhesion and invasion of gut epithelial cells. Other factors mediate biofilm formation and tolerance to low temperatures and high salt concentrations facilitating persistence and survival in the environment.

## Graphical abstract

*Listeria monocytogenes* is ubiquitous in both plant and animal reservoirs. It can persist in food production environments due to its capacity to grow at refrigerated temperatures and its resistance to biocides. The source of most human infections is contaminated food. Healthy individuals present with mild gastrointestinal symptoms. However, in immunocompromised individuals the infection is more severe, causing bacteraemia, meningitis and, in pregnancy-associated listeriosis, miscarriage and stillbirth. In vulnerable groups, including the elderly, pregnant women and their infants, listeriosis has a 20–30% mortality rate.

## Historical perspective

*Listeriamonocytogenes*, named after Joseph Lister, was isolated from a patient with meningitis in 1919, although it was first described in 1926 as a zoonotic pathogen affecting rabbits [1]. Listeriosis was identified as a foodborne illness in 1981 and is now recognized as an important biohazard in the food industry.

## Clinical presentation

Healthy individuals infected with *L. monocytogenes* experience mild gastroenteritis, such as nausea, diarrhoea and abdominal pain. However, in immunocompromised patients, including the elderly, those with underlying morbidities, and pregnant women and their infants, the *L. monocytogenes* can enter the bloodstream and become invasive. Patients with bacteraemia present with fever and chills. Complications include sepsis, meningitis, encephalitis and in pregnancy-associated listeriosis, miscarriage and stillbirth. In vulnerable patients, listeriosis has a mortality rate of 20–30% [2]. The incubation period is between 1–91 days.

## Microbial characteristics of phenotypic/genotypic features

*L. monocytogenes* is a Gram-positive, non-spore-forming, motile, facultatively anaerobic, rod-shaped bacterium with peritrichous flagella. It is an intracellular pathogen capable of surviving in macrophages. Based on phylogenetic analysis, *L. monocytogenes* is divided into four major lineages (I–IV). Serotypes within Lineage I (1/2a, 3b, and 4b) are more commonly associated from human cases, while Lineage II serotypes (1/2c and 3a) are more often recovered from food and food production environments. Lineages III and IV are associated with animals [3,4]. The ability to survive at low temperatures, high salt concentrations and low pH levels, and to form biofilms, facilitates survival and persistence in food and food processing environments [5]. *L. monocytogenes* is ubiquitous in nature, in the animal reservoir and in plant, water, and soil environments.

## Clinical diagnosis, laboratory confirmation and safety

### Clinical diagnosis

A clinical diagnosis is confirmed by the culture of *L. monocytogenes* from blood or cerebrospinal fluid, in patients presenting with sepsis or meningitis. Colonies are 0.5 to 1.5mm in diameter, smooth, translucent with a characteristic ground glass appearance able to be emulsified and with a zone of hazy β-haemolysis extending 1 to 2mm from the edge of the colony. Some strains of *L. monocytogenes* can be non-haemolytic. In pregnancy-associated listeriosis *L. monocytogenes* is cultured from vaginal swabs, placenta, meconium, or amniotic fluid.

### Laboratory confirmation

Under the microscopy, *L. monocytogenes* appears as Gram positive rods approximately 0.5×0.5 – 3 µm with rounded ends, occurring singly or sometimes in pairs and may resemble 'coryneforms' or diplococci. They are non-sporing, non-branching and non-capsulated. Preliminary identification tests include a positive catalase reaction, tumbling motility at 20–25 °C, β-haemolysis and aesculin hydrolysis. Historically, the confirmatory test was the Christie-Atkins-Munch-Peterson (CAMP) test, which can help confirm species by testing for haemolysis enhancement on sheep blood agar. In recent years, identification may be confirmed by MALDI-ToF or PCR and/or whole genome sequencing [6].

### Safety

*L. monocytogenes* is classified as a medium-risk biological organism and falls within Containment Level 2 (CL2) or Biosafety level 2 (BSL2) parameters. CL2/BSL2 laboratory protocols recommend that all bacterial manipulations be performed in a microbiological safety cabinet. Other interventions are focused on preventing injuries such as skin breakages, ingestion, and mucous membrane exposures. Pregnant women and immunocompromised staff are also strongly advised not to work with *L. monocytogenes* due to the potential for severe clinical outcomes in vulnerable groups.

## Treatment, resistance and evasion

### Treatment

Treatment regimens recommend amoxicillin, ampicillin or an aminoglycoside [7]. Listeriosis, manifesting as meningitis or septicaemia, requires intravenous amoxicillin/ampicillin. Trimethoprim-sulfamethoxazole (TMP-SMX) can be an alternative for penicillin allergy patients. For life-threatening infections, such as meningitis, gentamycin may be included.

### Resistance

Although antimicrobial resistance in *L. monocytogenes* is low, resistance to penicillin, ampicillin, tetracycline, macrolides, and fluoroquinolones linked to efflux pumps been reported. Resistance to various disinfectants, such as benzalkonium chloride, is common and enables *L. monocytogenes* to persist in many food production environments [7].

### Evasion

*L. monocytogenes* is an intracellular pathogen that can survive and replicate within host cells. The bacteria enter the host’s cells through phagocytosis/endocytosis. The bacteria escapes phagosome using listeriolysin O to replicate in the cytoplasm. More specifically, *L. monocytogenes* can highjack the host cells enzymes and compounds for actin production, which helps them propel from cell to cell, evading the immune system [8].

## Pathogenic strategies (host range, host response, transmission, infection and virulence factors)

### Host range

*L. monocytogenes* can transiently colonise the gut of humans and a wide range of animals, including cattle, sheep, goats, pigs, dogs, cats, domestic and wild rabbits, many other small mammals, and birds, including poultry. Clinical manifestations in animals include encephalitis, abortion, and sepsis [9].

### Host response

The innate immune response occurs within minutes of the bacteria entering the body, activating bacteria encasement and lymphocyte, limiting systemic spread, and reducing the risk of life-threatening complications. The acquired immunity response involving specific CD4+ and CD8+ T cells mediates complete clearance of infection [8]. Autophagy limits intracellular replication and pyroptosis of the infected cells limits the spread. Antibodies generated against *L. monocytogenes* can prevent reinfection.

### Transmission

Transmission of *L. monocytogenes* through the consumption of contaminated foods (such as deli meats, cold cuts, hot dogs, fermented or dry sausages, unpasteurized milk and milk products, refrigerated pate, meat spreads, smoked seafood and unwashed fruits and vegetables), and more rarely through contact with infected animals [9]. Vertical (mother-to-fetus) transmission is possible via the placenta during pregnancy and post-birth [2].

### Infection

Ingested *L. monocytogenes* infects survives in the human gut and penetrates gut mucosa into the bloodstream to spread systemically. Invasive listeriosis is associated with severe disease outcomes, including bacteraemia, and respiratory infections in elderly immunocompromised patients. Listeria can cross the blood-brain barrier leading to meningitis, encephalitis, and neurolisteriosis. Listeria can also cross the placental barrier with long-term effects on the unborn [2].

### Virulence factors

Strains associated with human disease are more likely to have Listeria Pathogenicity Islands 1&3 (LIPI-1) and LIPI-3, encoding genes responsible for invasive control, contributing to high virulence clones [8]. The listerial protein internalins mediate bacterial adhesion and invasion of epithelial cells in the human intestine through specific interaction with host cell receptors. The haemolysin, listeriolysin O (LLO) is a secreted pore-forming protein essential for the escape of *L. monocytogenes* from the vacuole formed upon initial internalization. ActA, an essential virulence factor of *Listeria monocytogenes*, is an integral membrane protein that is required for intracellular motility, cell-to-cell spread, and rapid dissemination of the bacteria in the infected host. Microbial phospholipases act as virulence factors triggering internalization into host cells and beyond that also the destruction of bacterial competitors [8].

Clones sampled from environmental, or food sources can survive in a multitude of environments, including a wide range of pH, high salt concentrations and refrigerated temperatures [9]. These clones often carry factors, like sigma (eg. σB) and transcription regulators, which deal with stress response, persistence and survival in food production environments [10].

## Epidemiology, prevention and risk groups

### Epidemiology

Foodborne listeriosis is a relatively rare disease with 0.1 to 10 cases per 1 million people per year depending on the countries and regions of the world. Although the number of notified cases of listeriosis is small, the high rate of death associated with this infection makes it a significant public health concern. The EU/EEA notification rate was 0.51 per 100 000 population (2268 confirmed cases). In the USA, an estimated 1 600 people get listeriosis each year, and there are approximately 260 fatalities. The most affected age group are those over 60 years, where a higher proportion of cases are male. Pregnancy-associated listeriosis contributes to the higher proportion of females in the younger age groups.

Outbreaks of listeriosis have been caused by a wide range of contaminate food items included contaminate dairy products, processed meat and fish products, and fresh and frozen vegetables [6,10].

### Prevention

*L. monocytogenes* is destroyed by pasteurization; heating foods to temperatures over 65 ^o^C inactivates the bacteria. Food factories and retailers systematically sample food aiming for its absence in 25 g and food contact surfaces. The European food safe limit level is up to 100 c.f.u. per g at any point in the shelf life of ready-to-eat products. However, *L. monocytogenes* can grow at refrigeration temperatures (−0.5–9.3 ^o^C), and it is essential to follow the “best by” dates. Cross contamination is common in cooking and food preparation areas, and disinfection and cleaning procedures are essential to avoid transmission [5].

High-risk-groups including pregnant women, immunocompromised patients, and people over 60 are advised to avoid processed ready-to-eat food, including unpasteurised products, soft cheeses, cured and smoked meats and seafood and consumption of raw packaged frozen vegetables [6,10].

### Risk groups

*L. monocytogenes* most commonly affects the people over 60 years old, the immunocompromised patients, pregnant women and their born and unborn infants.

## Open questions

1. What are the molecular mechanisms of pathogenicity in *L. monocytogenes*?
2. How and why do different serotypes of *L. monocytogenes* vary with respect to clincial outcomes, animal reservoirs, food sources and transmission routes?
3. How can we collect samples from mild cases of listeriosis?
4. Are we communicating risk appropriately and reaching all high-risk-groups?
5. How can we use whole genome sequencing data to enhance our understanding of virulence, infectivity and persistence?
